# Impact of ethical climate, moral distress, and moral sensitivity on turnover intention among haemodialysis nurses: a cross-sectional study

**DOI:** 10.1186/s12912-023-01212-0

**Published:** 2023-02-27

**Authors:** Haengsuk Kim, Hyunjung Kim, Younjae Oh

**Affiliations:** 1 Graduate School of Nursing Science, Hallym University, Hallymdaehakgil 1, 24252 Chuncheon, Gangwon-do Republic of Korea; 2 Kangdong Sacred Heart Hospital, Seongan-ro 150, Gangdong-gu, 05355 Seoul, Republic of Korea; 3 College of Nursing, Research Institute of Nursing Science, Hallym University, Hallymdaehakgil 1, 24252 Chuncheon, Gangwon-do Republic of Korea

**Keywords:** Turnover intention, Ethical climate, Moral distress, Moral sensitivity, Haemodialysis nurses

## Abstract

**Background:**

While several studies have been performed on turnover intention among nurses, limited studies have considered the ethical perspectives on turnover intention among haemodialysis nurses. The purpose of this study was to clarify the impact of ethical factors, namely ethical climate, moral distress, and moral sensitivity on turnover intention among haemodialysis nurses.

**Methods:**

This cross-sectional research was conducted between July and August 2017. A total of 148 haemodialysis nurses were invited to participate in the study by convenience sampling from 11 general and university hospitals in South Korea. Data were analysed using SPSS for t-test, one-way analysis of variance, Pearson’s correlation coefficients, and multiple regression analysis.

**Results:**

In the final regression model, the adjusted R-squared significantly explained 34.6% of the variance in turnover intention (F = 22.534, *p* < .001) when moral distress related to physician practice (β = 0.310, *p* = .001) and ethical climate related to the hospital climate (β = − 0.253, *p* = .003) and manager (β = − 0.191, *p* = .024) were included. Following the stepwise multiple regression process, all subdomains related to moral sensitivity were excluded due to no statistical significance in the final regression model.

**Conclusion:**

To reduce turnover intention among haemodialysis nurses, hospitals and managers should pay attention to haemodialysis nurses’ moral distress originating from physicians’ practice and improve their ethical climate. Additionally, it is required that the impact of moral sensitivity on turnover among nurses working in diverse care settings be examined further.

## Background

Nurses working in haemodialysis wards are often confronted with diverse ethical issues, particularly unexpected ethical problems, or dilemmas related to emerging high-biomedical technology. For instance, some patients may receive epochal treatment to prolong life with kidney replacement therapy or newly advanced biomedical information and technologies. However, this can cause unforeseen ethical problems related to futile care [1, 2], unwanted kidney donations due to social pressure [3], inequality in kidney transplants [4], and escalation in conflicts regarding the best treatment decision among patients, their families, and healthcare professionals [5, 6].

For nurses working in haemodialysis wards, it is further emphasized that they should play the role of moral agents that advocate human dignity and, simultaneously, recommend nursing perspectives based on ethical principles and norms [7, 8]. However, while resolving ethical issues, several nurses may fail to put their ethical beliefs into action due to their organizations’ situational constraints. Situational constraints in an organization include limitations in objective resources and those posed by the organizational climate, such as unethical leadership or hierarchical atmospheres, named ethical climate [9, 10].

Ethical climate is defined as a shared perception within the organization of what is ethically correct behaviour and how to deal with ethical issues to provide the basis and direction of members’ ethical behaviour [11]. Olson [11], a nursing scholar who addressed ethical climate in the healthcare context, analysed it as an ethical concept that reflected the ethical attributes of a hospital’s atmosphere. The ultimate goal of healthcare in hospital environments, unlike enterprises, was to protect and promote the physical and psychological well-being of human beings, rather than maximize productivity or profit. It has been reported that ethical climate influenced personal ethical standards for what nurses should do when faced with ethical problems and helped them identify ethical issues within their organization [9, 12]. Previous studies showed that when nurses were optimistic regarding the ethical climate of their organization, they trusted the ethical decisions of the organization, leaders, or other members [10, 13]. Consequently, nurses experienced less moral distress that resulted from organizational or situational factors [9, 13].

Moral distress refers to painful feelings or extreme psychological discomfort experienced by nurses in situations where they know the right things to perform, yet are forced to do the opposite of their ethical beliefs due to situational constraints, such as adverse ethical climates in hospitals or limitations of resources or nursing personnel [14]. When moral distress is not resolved and experienced repeatedly, nurses could experience psychological discomfort or symptoms, such as depression, powerlessness, headache, or hypertension [12, 13]. A recent systematic review on moral distress among nurses showed that numerous nurses experienced a moderate level of moral distress and were unable to cope well, which damaged their sustainability [15, 16]. Such distressful experiences that involve ethical issues threaten nurses’ professional commitment, and many consider changing their jobs or resigning [10, 17].

Meanwhile, moral sensitivity is significantly related to job engagement among professionals who have personally high ethical standards [9, 12]. Nurses are trained to be ethically sensitive and deliver nursing care that aligns with vulnerable people’s appeal through nursing education and self-reflection [18]. Nurses’ moral sensitivity is essential for achieving professional values that determine the maintenance of their careers. In recent qualitative studies, nurses who were sensitive to ethical issues felt distressed when compelled to compromise ethical beliefs and professional ethics in unethical situations, which ultimately hindered job continuity [13, 19]. Given the previous evidence, it is necessary to identify moral sensitivity as one of the influencing factors for turnover among nurses.

When faced with ethical problems, haemodialysis nurses may experience moral distress or uncertainty resulting from their organization’s ambiguous ethical standards [20, 21] or insufficient time and personnel to care for their patients, respectively [22, 23]. Moreover, repeated ethical difficulties may give rise to their professional and ethical integrity being at stake [21], and eventually lead to turnover intention [9, 13]. In other words, it was found that haemodialysis nurses could face myriad ethical problems that could threaten the continuity of their work. Although recent studies analysed factors that influenced turnover in haemodialysis nurses [24, 25], to the best of our knowledge, limited studies have considered ethical factors as threats to turnover intention. Given the context of the haemodialysis ward, where various ethical conflicts can occur, it is crucial to identify ethical factors that could affect turnover intention among haemodialysis nurses. Therefore, the purpose of this study was to identify the relationship between ethical factors (ethical climate, moral distress, and moral sensitivity) and turnover intention and their impact on turnover intention among haemodialysis nurses.

## Methods

### Research design

We adopted a cross-sectional design to fulfil the study objectives. Data were collected using questionnaires that were distributed to the nurses of the haemodialysis unit. They were asked to seal the questionnaires in an envelope for confidentiality. The questionnaires were collected by the researcher during hospital visits or by mail.

### Participants and settings

We recruited a convenience sample of 148 haemodialysis nurses from 11 general hospitals with more than 200 beds across South Korea between 19 July and 30 August 2017. All hospitals were equipped with a haemodialysis room capable of accommodating at least more than 20 patients. Since the average number of haemodialysis nurses was 7.73 nurses per general hospital [26], we collected nationwide data. Based on previous literature, novice nurses with less than one year of work experience were susceptible to the ethical climate [27, 28], and manager nurses experienced moral distress differently from staff nurses [10, 29]. Hence, we included staff nurses who currently worked in the haemodialysis wards, except for manager nurses, and had more than one year of work experience.

The statistical sample size was calculated considering a significance level of α = .05, power set at 80%, and medium effect size = .15 (two-tailed test). The number of relevant variables was determined using the G*power Program (3.1 version). Adding a dropout rate and error range of 20% resulted in a necessary statistical sample size of 148 nurses. Only 130 questionnaires were returned, with a response rate of 87.8%. After excluding seven questionnaires with ambiguous and missing responses, 123 questionnaires were included in the analysis.

### Instruments

#### General characteristics

The questions for measuring the participants’ general characteristics were based on previous studies [13, 17]. These were organized into eight items: gender, age, marital status, religion, educational level, position, years of nursing experience, and years of working in the current unit.

#### Turnover intention

The Korean version of Turnover Intention (KTI) was used, which was validated for nurses by Park [30] using the original instrument developed by Lawler [31]. Permission to use the KTI was granted by Park [30]. The KTI is a four-item scale with no subdomains, each rated on a 5-point Likert scale that ranges from 1 (none) to 5 (severe). A higher score indicates a higher degree of turnover intention. Lawler [31] and Park [30] reported Cronbach’s alpha reliabilities of 0.83 and 0.88, respectively. Our study supported the reliability of the KTI with Cronbach’s α = 0.89.

#### Ethical climate

The Korean version of the Hospital Ethical Climate Survey (K-HECS) was used, which was validated by Hwang, Park [32] using the HECS created by Olson [11]. Permission to use the K-HECS was granted by Olson [11] and Hwang, Park [32]. The K-HECS is a 26-item scale that consists of the perception of ethical attitudes and behaviours in five subdomains, namely: “peers (4 items),” “patients (4 items),” “managers (6 items),” “hospital (6 items),” and “physicians (6 items).” Participants were asked to score each item on a 5-point Likert scale that ranged from 1 (almost never true) to 5 (almost always true). A higher score indicates a more positive perception of the ethical climate. Olson [11] and Hwang, Park [32] reported Cronbach’s alpha reliabilities of 0.91 and 0.95, respectively. Our study supported the reliability of the K-HECS with a Cronbach’s α = 0.93.

#### Moral distress

The Korean version of the Moral Distress Scale-Revised (KMDS-R) validated by Chae et al. [33] using the Moral Distress Scale-Revised (MDS-R) developed by Hamric et al. [34], was used. Permission to use the KMDS-R was granted by Hamric and Chae. The KMDS-R is a 21-item scale comprising the situations that cause moral distress in five subdomains for “futile care (5 items),” “nursing practice (5 items),” “institutional and contextual factor (4 items),” “physician practice (4 items),” and “limit to claim the ethical issue (3 items).” Responses were rated on of a 5-point Likert scale developed to measure the frequency of moral distress (0: *“never”* to 4: *“very frequently”*) and the intensity of moral distress (0: *“none”* to 4: *“great extent”*). The moral distress score was calculated by multiplying the frequency and intensity scores of distress by “0” if one had never experienced or reported no intensity in any item. The number of points of moral distress was calculated as the total score by adding all the scores for each question. A higher score indicated a stronger sense of moral distress. Hamric et al. [34] and Chae et al. [33] reported Cronbach’s alpha reliabilities of 0.89 and 0.91, respectively. This study supported the KMDS-R’s reliability with a Cronbach’s α = 0.85.

#### Moral sensitivity

The Korean version of the Moral Sensitivity Questionnaire (K-MSQ) was used, which was validated by Han et al. [35] using the original version, MSQ by Lützén et al. [36]. Lützén and Han granted permission to use the K-MSQ. The K-MSQ is a 27-item scale that includes two reverse-coded items. It comprised five subcategories of “patient-centred nursing care (5 items)”, “professional responsibility (7 items)”, “conflicts (5 items)”, “moral meaning (5 items)”, and “beneficence (5 items).” Responses were rated on a 7-point Likert scale that ranged from 1 (strongly disagree) to 7 (strongly agree). The score ranged from 27 to 189: the higher the score, the higher the moral sensitivity. Han et al. [35] found the scale’s reliability to be 0.89. In our study, Cronbach’s α was 0.86.

#### Data analysis

The normal distribution of the data was confirmed by the Shapiro-Wilk test (*p* > .05). Cronbach’s alpha reliability analysis, t-test, one-way analysis of variance (ANOVA), Pearson’s correlation coefficients, and multiple regression analysis were conducted using SPSS for Windows version 21.0.

#### Ethical considerations

The study was conducted in accordance with the Declaration of Helsinki and approved by the Institutional Review Board of Hallym University before data collection (HIRB NO. 2017-040). Written informed consent regarding the study purpose, which included guaranteed anonymity and confidentiality, was obtained. Only those who voluntarily agreed to participate were sampled, and participants could withdraw at any time without repercussions. When participants could not deliver the questionnaire to the researcher directly due to their work shift, they were asked to seal it in an envelope for confidentiality.

## Results

### General characteristics and differences in turnover intention according to the general characteristics

Of all the participants, 43.9% were aged 30–39 years, with a mean age of 35.45 years (SD = 7.60). Most were female (96.7%), had bachelor’s/graduate degrees (82.1%), and over half had no religion (57.7%). The mean years of nursing experience and experience in a current unit were 13.15 (SD = 9.75) and 6.65 years (SD = 6.17), respectively. There were no differences in turnover intention according to the general characteristics (Table 1).

**Table 1:** General characteristics and differences in turnover intention according to the general characteristics (n = 123)

Characteristics	Categories	n (%) or mean (SD)		Turnover Intention			
				Mean	(SD)	t or F	*p*
Gender	Female	119	(96.7)	3.61	(1.02)	-1.228	.222
	Male	4	(3.3)	4.25	(.96)		
Age(year)	20–29	29	(23.6)	3.77	(1.04)	1.125	.342
	30–39	54	(43.9)	3.65	(1.06)		
	40–49	34	(27.6)	3.40	(.99)		
	50 and older	6	(4.9)	4.08	(.72)		
		35.45	(7.60)				
Marital status	Married	43	(35.0)	3.77	(.91)	1.074	.285
	Unmarried	80	(65.0)	3.56	(1.08)		
Religion	With a religion	52	(42.3)	3.68	(.98)	.467	.642
	Without a religion	71	(57.7)	3.60	(1.07)		
Educational level	Associate degree	22	(17.9)	4.10	(.79)	2.944	.056
	Bachelor degree	85	(69.1)	3.52	(1.09)		
	Graduate school	16	(13.0)	3.59	(.80)		
Position	Staff nurse	112	(94.1)	3.67	(1.05)	.499	.618
	Chief nurse	7	(5.9)	3.46	(.73)		
Years of nursing experience	1 - < 3 years	18	(14.9)	3.71	(1.16)	.560	.731
	3 - < 5 years	6	(5.0)	3.96	(.73)		
	5 - < 10 years	21	(17.4)	3.70	(.99)		
	10 - < 15 years	28	(23.1)	3.71	(1.02)		
	15 - < 20 years	21	(17.4)	3.48	(1.24)		
	> 20 years	27	(22.3)	3.40	(.87)		
		13.15	(9.75)				
Years of working at a current unit	1 - < 3 years	43	(35.8)	3.75	(.97)	1.076	.378
	3 - < 5 years	19	(15.8)	3.39	(1.20)		
	5 - < 10 years	22	(18.3)	3.85	(1.04)		
	10 - < 15 years	21	(17.5)	3.55	(.99)		
	15 - < 20 years	11	(9.2)	3.18	(.99)		
	> 20 years	4	(3.3)	3.25	(.79)		
		6.65	(6.17)				

### Levels of turnover intention, ethical climate, moral distress, and moral sensitivity

The mean turnover intention and ethical climate were 3.63 (SD = 1.03) and 96.24 (SD = 10.05), respectively. In the five subdomains, the highest and lowest means were for the subdomains “peers” (mean = 4.20, SD = .50) and “hospital” (mean = 3.35, SD = .48), respectively. The mean moral distress was 98.55 (SD = 66.24). In the five sub-dimensions, the highest and lowest means were for “futile care” (mean = 6.69, SD = 4.09) and “limit to claim the ethical issue” (mean = 2.08, SD = 2.49), respectively. The mean score for moral sensitivity was 4.88 (SD = .57). In the five subdomains, the highest and lowest means were for the “professional responsibility” (mean = 5.46, SD = .72) and “benevolence” (mean = 4.02, SD = .79; Table 2), respectively.

**Table 2:** Levels of turnover intention, ethical climate, moral distress, and moral sensitivity (n = 123)

Variables			Mean (SD)	Range	Min-Max
Turnover intention			3.63(1.03)	1–5	1.00–5.00
Ethical climate			96.24(10.05)	26–130	69.00-123.00
		Peers	4.20(0.50)		3.00–5.00
		Managers	4.13(0.61)		2.00–5.00
		Patients	3.77(0.42)		2.60–6.20
		Physicians	3.34(0.67)		1.67-5.00
		Hospital	3.35(0.48)		2.17–4.33
Moral distress			98.55(66.24)	0-336	7.00-264.00
		Futile care	6.69(4.09)		0.00–16.00
		Nursing practice	5.30(4.64)		0.00–16.00
		Institutional and contextual factors	5.01(4.03)		0.00–15.00
		Physician practice	3.08(2.77)		0.00–11.00
		Limit to claim the ethical issue	2.08(2.49)		0.00-11.33
Moral sensitivity			4.88(0.57)	1–7	3.37–6.56
		Professional responsibility	5.46 (0.72)		3.29-7.00
		Patient-oriented care	5.43 (0.71)		3.40-7.00
		Conflict	4.72 (0.82		2.60-7.00
		Moral meaning	4.54 (0.82		2.20–6.80
		Benevolence	4.02 (0.79)		2.60–6.80

### Correlations among turnover intention, ethical climate, moral distress, and moral sensitivity

Turnover intention was negatively and positively correlated with ethical climate (r = − .549, *p* < .001) and moral distress (r = .456, *p* < .001), respectively. The correlations between all the subdomains of ethical climate and turnover intention were significant, except for the “patients.” The correlations between all the subdomains of moral distress and turnover intention were significant, except for “limit to claim the ethical issue” and “futile care.” There was no correlation between turnover intention and moral sensitivity (see Table 3).

**Table 3:** Correlation among turnover intention, ethical climate, moral distress, and moral sensitivity (n = 123)

		Turnover Intention		
		r	*p*	
Ethical climate		− 0.549	< 0.001	
	Hospital	− 0.469	< 0.001	
	Physicians	− 0.447	< 0.001	
	Managers	− 0.422	< 0.001	
	Peers	− 0.350	< 0.001	
	Patients	− 0.115	0.203	
Moral distress		0.456	< 0.001	
	Physician practice	0.526	< 0.001	
	Nursing practice	0.421	< 0.001	
	Institutional and contextual factor	0.421	< 0.001	
	Limit to claim the ethical issue	0.309	0.001	
	Futile care	0.268	0.003	
Moral sensitivity		0.012	0.891	
	Patient-centred care	0.046	0.601	
	Professional responsibility	− 0.076	0.394	
	Conflict	0.132	0.137	
	Benevolence	0.038	0.668	
	Moral meaning	− 0.073	0.412	

### Impact of ethical climate and moral distress on turnover intention

This section identified the impact of ethical climate, moral distress, and moral sensitivity on turnover intention using a stepwise multiple regression analysis. Following the stepwise multiple regression process, all subdomains related to moral sensitivity were excluded due to no statistical significance. In the final regression model, the adjusted R-squared was significant, which explained 34.6% of the variance of turnover intention (F = 22.534, *p* < .001), when “physician practice” (*β* = 0.310, *p* = .001) from the moral distress and “hospital” (*β* = − 0.253, *p* = .003) and “manager” (*β* = − 0.191, *p* = .024) from the ethical climate were included (Table 4). The stepwise regression model was evaluated for multicollinearity. The Durbin–Watson statistic was 1.736, close to 2.0, indicating no autocorrelation in the residuals. The variance inflation factor (VIF) was 1.314–1.549 (smaller than 10) and did not present multicollinearity concerns [37].

**Table 4:** Impact of ethical climate and moral distress on turnover intention (n = 123)

Independent Variables		Unstandardized Coefficients		Standardized Coefficients			Collinearity Statistics	
		B	S.E.	β	t	*P*	Tolerance	VIF
(constant)		6.437	0.862		7.466	< 0.001		
Moral distress								
	Physician practice	0.115	0.034	0.310	3.403	0.001	0.646	1.549
Ethical climate								
	Hospital	− 0.546	0.183	− 0.253	-2.981	0.003	0.745	1.342
	Manager	− 0.322	0.141	− 0.191	-2.280	0.024	0.761	1.314

*Note*: F = 22.534, *p* < .001, *R*^*2*^ = 0.362, Adjusted *R*^*2*^ = 0.346, *d(du)* = 1.736

## Discussion

In this study, moral distress caused by physician practice was a significant factor in turnover intention among haemodialysis nurses. It was challenging to compare this finding with previous results since studies on the impact of moral distress on turnover among nurses reported a single continuum [38, 39] or used different instruments to assess moral distress [40, 41]. Nevertheless, some findings were comparable and consistent with ours, which reported that nurses felt ethically distressed when they could not take the right action due to lack of authority, even though they perceived inappropriate ethical behaviours or attitudes by physicians toward patients [10, 13, 29]. Furthermore, the nursing environment was dominated by medical paternalism and hierarchism in South Korea, which led numerous nurses to consider changing or leaving their job [13, 29]. In cultural contexts or hierarchical power structures similar to that of hospitals in South Korea, medical paternalism gave rise to moral distress among nurses in Taiwan [42] and Iran [43] or hindered their professional commitment in China [44]. Moral distress and professional commitment among nurses are well-known factors that majorly contribute to turnover.

According to Fischer Grönlund et al. [23], haemodialysis nurses sometimes differed from physicians’ treatment plans since they strived to advocate following patients’ perspectives and focused on patients’ narratives. For instance, therapeutic relationships between patients and nurses have gradually advanced in haemodialysis wards. Good nursing care can be achieved by person-centred care [45]; in the 3–4 hours during haemodialysis, nurses are able to attentively listen to unique stories from individual patients. Furthermore, most patients visit the haemodialysis room regularly for a long time, such as once or more every week for several years [46, 47]. However, nurses cannot appropriately participate in the treatment decision-making process. Haemodialysis nurses can feel psychological discomfort, such as powerlessness, helplessness, or despair, regarding the physicians’ unethical actions that threaten their nursing values to advocate for the patients [25, 48]. In previous studies, diminished nursing values were found to impede professional commitment and cause turnover intention [49, 50]. Although many quantitative studies focused on the association between moral distress and turnover intention in nursing, further qualitative studies should explore the essence of turnover intention in situations where nurses were confronted with moral distress that resulted from physician practice.

Our findings support previous studies that showed that turnover intention was negatively associated with nurses’ perceptions of ethical climate [51, 52]. Particularly, a negative perception of the hospital’s ethical climate influenced turnover intention among haemodialysis nurses. This could be explained by the person–environment fit model [53], related to the fit between the person and environment, that suggests environmental factors could potentially impact employee turnover. A higher person-environment fit promotes an employee’s work state and establishes the primary premise and management foundation for building trustworthiness between employees and the organization [54]. Therefore, considering work environments in nursing, nurses reported the changing hospitals’ unethical attitude as the primary agenda for a safe and healthy work environment [9, 17]. According to research on ethical conflicts, nurses highlighted concerns, such as the hospital management viewing their patients as “customers” who could produce economic benefits, as well as hospitals’ punitive actions towards courageous ethical behaviours, such as providing negative feedback on nurses’ whistle-blowing [13]. Inadequate organizational attitudes and behaviours have been identified as factors that contribute to demoralization among haemodialysis nurses and can impact the quality of nursing care [23, 55].

In contrast, a recent scoping review on turnover among nurses summarized that nurses perceived their work environment as ethical; however, most included articles originated from Western countries, such as the United States or the United Kingdom [52]. Since cultural and individual contexts can significantly influence the ethical climate perceived by nurses, a further literature reviews on the association between ethical climate and turnover intention are required to integrate findings from diverse cultural backgrounds.

Another significant factor that influenced turnover intention among haemodialysis nurses was the perception of nurse managers’ ethical attitudes and behaviours. This finding was consistent with those of previous studies on nurses in other care settings [52, 56]. This consistent phenomenon can be explained by the social learning theory— according to which the followers copied the behaviours of their leader, who showed normative and trustworthy behaviours with reliable and ethical standards. Establishing clear ethical standards for leaders involves promoting normatively appropriate conduct through role modelling and interpersonal relationships. Managers’ ethical attitudes and behaviours can encourage employees to be more devoted to their companies by fostering a positive ethical climate. This leads employees ready to counsel ethical concerns with their manager and decreases their withdrawal condition [57]. A recent qualitative study on moral injury among haemodialysis nurses [48] showed that nurses emphasized the need for managers to discuss ethical concerns and establish imperatives. When confronted with ethical situations, unsupportive leaders could result in moral injury among nurses, resulting in an escalation of their psychological discomfort, including moral distress, leading to them considering leaving. In contrast, managerial ability, leadership, and support of nurses were not significantly associated with turnover among haemodialysis nurses [55]. Further studies should identify the impact of nurse leaders’ ethical attitudes and behaviours or ethical leadership on turnover intention among haemodialysis nurses and explore in-depth the experiences of how nurses perceive their nurse leaders’ role in ethical situations.

Meanwhile, there was no correlation between turnover intention and moral sensitivity. Nevertheless, our study’s hypothesis was based on previous evidence. For instance, nurses with higher moral sensitivity had significantly higher job stress and anxiety, which threatened job engagement and caused turnover [58]. Moreover, Ohnishi et al. [59] insisted that it was essential to prevent nurses with high moral sensitivity from burnout or turnover. Further research is required to verify the relationship between moral sensitivity and turnover in various work environments and to clarify the impact of high moral sensitivity on turnover among nurses.

### Implications

This study had several strengths. First, our study verified ethical climate and moral distress as factors that contributed to turnover intention in the context of haemodialysis nursing. Empirically, threatening nurses’ moral integrity was confirmed as a cause of turnover intention among haemodialysis nurses in their professional careers. Second, the main factor that influenced turnover intention was identified to gain insights into haemodialysis nurses’ moral distress, which resulted from ethically inappropriate physician practice. Third, our study highlights the adverse outcomes resulting from a vulnerable work environment where a hospitals’ and the managers’ ethical attitudes and behaviours are perceived as unethical. In this regard, hospitals and nurse managers should improve their ethical climate to motivate nurses to value their work and improve job retention and positive contributions to patient outcomes. Finally, our findings can serve as ideas or strategies to establish an ethics program or research or nursing management system to alleviate haemodialysis nurses’ moral distress, which can contribute to reducing turnover.

### Limitations

Our study had some limitations. First, convenience sampling was employed, resulting in weak generalizability. However, our data were collected from diverse work environments due to the limited number of nurses who worked in each hospitals’ haemodialysis unit [26]. Second, most participants were female since male Korean nurses accounted for only 5.1% of registered nurses in 2019 [26], implying that gender equity and gender differences may not be accurately represented. Further research is required to illuminate ethical factors related to turnover intention among male nurses and achieve gender equity based on standards, such as the Sex and Gender Equity in Research (SAGER) guidelines [60]. Third, data were obtained using self-report questionnaires. Some participants may have underreported their responses to moral distress since they may be targeted by their hospitals through disadvantaged feedback grounds of defaming their organizations. Some nurses were reluctant to join the research on ethical issues in their hospitals in other studies [13]. However, this concern can be reduced by employing psychometrically sound measures [61].

## Conclusion

Turnover intention among haemodialysis nurses was significantly influenced by moral distress that resulted from physicians’ practice and unethical climate related to their hospitals and managers. Although the impact of moral sensitivity on turnover in our study was found to be not significant, further research on it is recommended in the context of diverse care settings. To reduce turnover intention among haemodialysis nurses, it is vital to alleviate their moral distress and improve the hospitals’ ethical climate. Notably, it is suggested to pay attention to the nurses’ moral distress involved in physician practice and hospitals and nurse managers should improve the ethical climate so that haemodialysis nurses can maintain their job continuity.

## Data Availability

Data are available upon request from the corresponding author of the study.
